# The Modified Glasgow Prognostic Score as a Predictor of Survival After Hepatectomy for Colorectal Liver Metastases

**DOI:** 10.1245/s10434-013-3342-6

**Published:** 2014-01-23

**Authors:** Kazuya Nakagawa, Kuniya Tanaka, Kazunori Nojiri, Takafumi Kumamoto, Kazuhisa Takeda, Michio Ueda, Itaru Endo

**Affiliations:** 1 Department of Gastroenterological Surgery, Yokohama City University Graduate School of Medicine, Yokohama, Japan; 2 Department of Surgery, Gastroenterological Center, Yokohama City University, Yokohama, Japan

## Abstract

**Background:**

The inflammation-based Glasgow prognostic score (GPS) has been demonstrated to be prognostic for various tumors. We investigated the value of the modified GPS (mGPS) for the prognosis of patients undergoing curative resection for colorectal liver metastases (CRLM).

**Methods:**

A total of 343 patients were enrolled onto this study. The mGPS was calculated as follows: mGPS-0, C-reactive protein (CRP) ≤10 mg/L; mGPS-1, CRP >10 mg/L and albumin ≥35 g/L; and mGPS-2, CRP >10 mg/L and albumin <35 g/L. Prognostic significance was retrospectively analyzed by univariate and multivariate analyses.

**Results:**

Of the 343 patients, 295 (86.0 %) were assigned to mGPS-0, 33 (9.6 %) to mGPS-1, and 15 (4.4 %) to mGPS-2. The median disease-free survival of patients with mGPS-0, -1, and -2 was 18.3, 15.5, and 5.2 months, respectively. The median cancer-specific survival (CSS) of patients with mGPS-0, -1, and -2 was 89.5, 62.2, and 25.8 months, respectively. The CSS of patients with mGPS-0 was significantly longer than that of patients with mGPS-2. Multivariate analysis revealed a significant association between cancer-related postoperative mortality and mGPS and carcinoembryonic antigen level.

**Conclusions:**

The preoperative mGPS is a useful prognostic factor for postoperative survival in patients undergoing curative resection for CRLM.

Colorectal cancer (CRC) is the third most common cancer worldwide, with a cumulative lifetime risk of ~5 %.^1^,^2^ Despite improvements in hepatectomy techniques (e.g., aggressive liver resection including major vessel resection and two-stage hepatectomy combined with chemotherapy) and introduction of new postoperative chemotherapy regimens, overall survival is still poor for most patients with colorectal liver metastases (CRLM).^3^–^6^ 5-Year survival rates after hepatic resection reportedly range from 33 to 61 %.^7^–^13^

There is increasing evidence that the presence of an ongoing systemic inflammatory response, as revealed by an elevated concentration of circulating serum C-reactive protein (CRP), is associated with poor outcomes in patients with advanced cancers.^14^–^20^ Recent studies have revealed that the Glasgow prognostic score (GPS), an inflammation-based prognostic score that includes only serum CRP and serum albumin, is one of the most useful scoring systems for the prognostication of patients with advanced cancer.^21^–^28^ Several studies have investigated the value of the GPS for postoperative prognostication of patients undergoing curative resection for CRC.^29^,^30^ However, few studies have reported the GPS in patients with CRLM who underwent liver resection.^31^,^32^

The GPS was recently modified on the basis of evidence that hypoalbuminemia in patients without an elevated CRP concentration has no significant association with cancer-specific survival (CSS).^30^ There is a considerable body of evidence supporting that the modified GPS (mGPS) can predict CSS in patients undergoing curative resection for CRC.^33^–^35^ To our knowledge, no study has investigated the usefulness of the mGPS in patients undergoing liver resection for CRLM. Therefore, the aim of this study was to evaluate the value of the mGPS for prediction of postoperative death in patients with CRLM.

## Materials and Methods

### Patients

Between January 1988 and December 2010, a total of 433 patients with CRLM underwent initial liver resection at university-affiliated hospitals (Graduate School of Medicine, Yokohama City University, and Yokohama City University Medical Center). Of these, 75 were excluded from analysis because extrahepatic disease was present at surgery, 14 were excluded because data on their CRP and albumin levels were not available, and 1 was excluded because of postoperative death (within 30 days). The remaining 343 patients were enrolled onto this study. None of the patients exhibited clinical evidence of infection or any other inflammatory conditions. The median follow-up period for survivors was 54.4 months (range 2–237 months). The mGPS was estimated as described previously.^33^ Briefly, patients with an elevated CRP level (>10 mg/L) were allocated as mGPS-1 or -2 depending on the absence or presence of hypoalbuminemia (<35 g/L), and patients with no elevation of CRP (≤10 mg/L) were allocated as mGPS-0. The extent of the resection was recorded as major or minor, with a major resection defined as a resection of more than three segments.

### Patient Follow-Up

Patients were followed up monthly at our outpatient clinic. Data were obtained and recorded, and long-term outcomes were determined through clinical follow-up, cancer registry follow-up, and contact with the patient, family, or referring physician when necessary. Serum CEA levels were measured monthly, and computed tomography (CT) was performed every 3 months. Recurrence was defined as a lesion that was biopsy-proven recurrent adenocarcinoma or a lesion that was deemed suspicious on cross-sectional imaging in the setting of an elevated CEA level. The end of follow-up was the time of last follow-up (March 2012) or death.

### Adjuvant Therapy

After resection of liver metastases or extrahepatic metastases, adjuvant chemotherapy was carried out generally via hepatic artery infusion or by intravenous infusion, usually with 5-fluorouracil and l-folinic acid with or without the addition of oxaliplatin or irinotecan. In all patients who received prehepatectomy chemotherapy, the same preoperative chemotherapy was usually continued postoperatively as adjuvant therapy.

### Statistical Analysis

Data are presented as mean ± standard error (SE). Differences between groups were analyzed using the Mann–Whitney *U* test and *χ* ^2^ test. Hazard ratios (HR) with 95 % confidence intervals (95 % CI) were calculated using univariate or multivariate analysis.

Survival was measured from the time of hepatic resection to death, which was the end point. Survival curves were constructed by the Kaplan–Meier method and compared using a log-rank test. Statistical significance was defied as *P* < 0.05. Analysis was performed using the software package Dr. SPSS II (SPSS Inc., Chicago, IL, USA).

## Results

### Relationships Between mGPS and Patient Characteristics

Among the 343 patients, 48 (14.0 %) had an elevated CRP level (>10 g/L) and 29 (8.5 %) had hypoalbuminemia (<35 g/L). Fifteen (4.4 %) patients had both elevated CRP and hypoalbuminemia. Of the 343 patients, 295 (86.0 %) were assigned to mGPS-0, 33 (9.6 %) to mGPS-1, and 15 (4.4 %) to mGPS-2.

Patient characteristics in each mGPS group are provided in Table 1. Patients with mGPS-2 were older than those with mGPS-0 (*P* = 0.046) or 1 (*P* = 0.036). The maximum tumor size was larger in patients with mGPS-1 (*P* = 0.006) or 2 (*P* = 0.030) than in those with mGPS-0. Patients with mGPS-1 or 2 had higher levels of white blood cells (mGPS-0 vs. 1, *P* = 0.001; mGPS-0 vs. 2, *P* < 0.001), neutrophils (mGPS-0 vs. 1, *P* < 0.001; mGPS-0 vs. 2, *P* < 0.001), and CEA (mGPS-0 vs. 1, *P* = 0.036; mGPS-0 vs. 2, *P* = 0.005) than those of patients with mGPS-0. There were no significant differences between mGPS and patient characteristics such as gender, primary site (colon/rectum), timing (synchronous/metachronous), distribution (unilobar/bilobar), or tumor number.

**Table 1:** Relationship between mGPS and clinical background

Characteristic	mGPS-0 (*n* = 295)	*P* value, mGPS-0 vs. 1	mGPS-1 (*n* = 33)	*P* value, mGPS-1 vs. 2	mGPS-2 (*n* = 15)	*P* value, mGPS-0 vs. 2
Age	63.7 ± 0.6	0.495	52.0 ± 1.7	0.036	69.5 ± 2.7	0.046
Gender						
Male	191 (65 %)	0.826	22 (67 %)	0.082	6 (40 %)	0.052
Female	104 (35 %)		11 (33 %)		9 (60 %)	
Site of primary lesion						
Colon	166 (57 %)	0.953	19 (58 %)	0.482	7 (47 %)	0.429
Rectum	125 (43 %)		14 (42 %)		8 (53 %)	
Histologic differentiation						
Moderate	179 (63 %)	0.020	14 (42 %)	0.119	10 (67 %)	0.789
Other	104 (37 %)		19 (58 %)		5 (33 %)	
Timing						
Synchronous	144 (49 %)	0.052	22 (67 %)	0.746	11 (63 %)	0.110
Metachronous	151 (51 %)		11 (33 %)		4 (27 %)	
Distribution						
Unilobar	178 (60 %)	0.328	17. (52 %)	0.907	8 (53 %)	0.589
Bilobar	177 (40 %)		16 (48 %)		7 (47 %)	
No. of liver metastases	3.3 ± 0.2	0.094	5.5 ± 1.1	0.262	3.5 ± 1.3	0.960
Single	131 (44 %)	0.582	13 (39 %)	0.636	7 (47 %)	
Multiple	164 (56 %)		20 (61 %)		8 (53 %)	0.864
Maximum tumor size, cm	33.3 ± 1.4	0.006	47.9 ± 5.5	0.954	48.5 ± 8.0	0.030
Extent of liver resection						
Minor	202 (68 %)	<0.001	12 (36 %)	0.053	10 (67 %)	0.883
Major	93 (32 %)		21 (64 %)		5 (33 %)	
Perioperative chemotherapy						
Neo-adjuvant	62 (21 %)	0.979	7 (21 %)	0.056	0	0.047
Adjuvant						
Systemic	60 (20 %)		7 (21 %)		1 (7 %)	
HAI	75 (25 %)	0.679	11 (33 %)	0.202	6 (40 %)	0.284
Systemic + HAI	49 (17 %)		4 (12 %)		3 (20 %)	
Preoperative laboratory data						
White blood cell	5,600 ± 99	0.001	6,900 ± 402	0.081	7,900 ± 528	<0.001
Neutrophil	3,300 ± 84	<0.001	4,400 ± 331	0.052	5,300 ± 403	<0.001
Lymphocyte	1,600 ± 35	0.684	1,600 ± 125	0.584	1,432 ± 117	0.280
CEA, ng/L	88 ± 22	0.036	316 ± 160	0.268	418 ± 228	0.005

*mGPS* modified Glasgow prognostic score, *HAI* hepatic arterial infusion, *CEA* carcinoembryonic antigen

### Recurrence and Recurrence Pattern

**Fig. 1:**
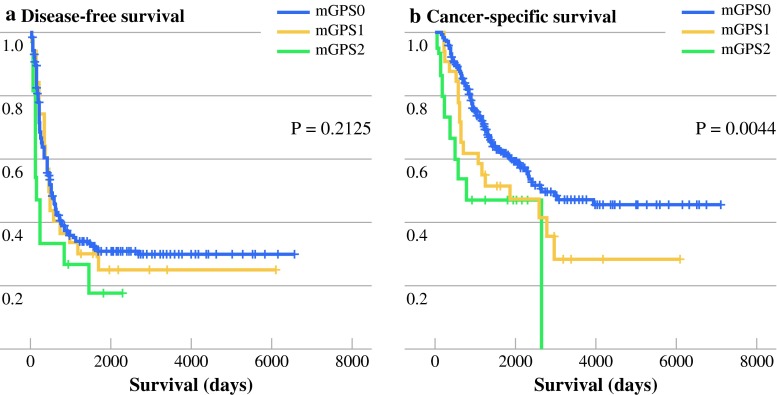
Relationship between mGPS (0, 1, and 2 from top to bottom) and disease-free survival in patients with CRLM (**a**). Relationship between mGPS (0, 1, and 2 from top to bottom) and cancer-specific survival in patients with CRLM (**b**)

Overall, 224 (65.3 %) patients experienced recurrence of disease at the time of last follow-up. The number of patients with recurrence in the mGPS-0, -1, and -2 groups was 190 of 295 (64.4 %), 22 of 33 (66.7 %), and 12 of 15 (80.0 %), respectively. The median DFS of patients with mGPS-0, -1, and -2 was 18.3, 15.5, and 5.2 months, respectively. Kaplan–Meier analysis and the log-rank test demonstrated no significant differences among patients with mGPS-0, -1, and -2 (*P* = 0.2125) (Fig. 1).

The initial recurrence pattern is summarized in Table 2. The initial recurrence pattern was liver only in 84 (24.5 %) patients, lung only in 61 (17.8 %), other single sites in 43 (12.5 %), and multiple organ sites in 34 (9.9 %). There were no significant differences in the initial recurrence pattern between mGPS-0, -1, and -2 (*P* = 0.126). We focused on the initial liver-only recurrence, and the numbers of recurrent tumors among the mGPS groups are summarized in Table 3. The number of patients with liver-only recurrence in the mGPS-0, -1, and -2 groups was 72, 8, and 4, respectively. The number of patients with multiple liver recurrences (≥4) in the mGPS-0, -1, and -2 groups was 19 (26.4 %), 3 (37.5 %), and 4 (100 %), respectively. The number of patients with multiple liver recurrences in the mGPS-0 group (19 of 72) was significantly smaller than that in the mGPS-1 and 2 groups (7 of 12; *P* = 0.03).

**Table 2:** Relationship between mGPS and initial recurrence pattern

Site of recurrence	mGPS-0	mGPS-1	mGPS-2
	(*n* = 190)	(*n* = 22)	(*n* = 12)
Liver only	72 (38 %)	8 (36 %)	4 (33 %)
Lung only	50 (26 %)	8 (36 %)	3 (25 %)
Other single sites	36 (19 %)	3 (14 %)	4 (33 %)
Multiple organ sites	30 (16 %)	3 (14 %)	1 (9 %)
Unknown	2 (1 %)	0	0

*mGPS* modified Glasgow prognostic score

**Table 3:** Relationship between mGPS and number of tumor recurrences in patients with initial liver-only recurrence

No. of tumor recurrences	mGPS-0	mGPS-1	mGPS-2
	(*n* = 72)	(*n* = 8)	(*n* = 4)
1	31 (43 %)	3 (37.5 %)	0
2	13 (18 %)	0	0
3	9 (13 %)	2 (25 %)	0
≤4	19 (26 %)	3 (37.5 %)	4 (100 %)
Resection	34 (47 %)	0	1 (25 %)

*mGPS* modified Glasgow prognostic score

### Resection for Liver or Lung Recurrence

Among the 84 patients with liver recurrence, 35 (41.7 %) underwent resection. Resection for initial liver recurrence was performed in 34 (47.2 %) patients with mGPS-0, in no patients with mGPS-1, and in 1 (25.0 %) patient with mGPS-2 (Table 3). The rate of resected liver recurrence was significantly higher in patients with mGPS-0 than in those with mGPS-1 and 2 (*P* < 0.01). Radiofrequency ablation (RFA) was performed in two (2.8 %) patients with mGPS-0, in two (25.0 %) with mGPS-1, and in none with mGPS-2. The rate of treatment for local control, including liver resection and RFA, was also significantly higher in patients with mGPS-0 than in those with mGPS-1 and 2 (*P* < 0.01).

On the other hand, among 82 patients with lung recurrence, 27 (32.9 %) underwent resection. Resection for initial lung recurrence was performed in 22 (31.9 %) patients with mGPS-0, in 5 (55.6 %) with mGPS-1, and in none with mGPS-2. The rate of resected lung recurrence tended to be higher in patients with mGPS-0 or 1 than in those with mGPS-2, but there was no significant difference among the three groups (*P* = 0.133).

### Survival

Overall, 94 (27.4 %) patients died during this study. Of these, there were eight non-cancer-related deaths. The median CSS of patients with mGPS-0, -1, and -2 was 89.5, 62.2, and 25.8 months, respectively. Kaplan–Meier analysis and the log-rank test demonstrated significant differences among patients with mGPS-0, -1, and -2 (*P* = 0.0044), with a higher cancer-related mortality rate in patients with a higher mGPS (Fig. 1). Kaplan–Meier analysis and the log-rank test demonstrated that patients with mGPS-0 survived longer than those with mGPS-2 (*P* = 0.0040). Furthermore, survival of patients with mGPS-1 tended to be poorer than that of patients with mGPS-0 (*P* = 0.0584).

### Prognostic Factors

Univariate analysis of postoperative mortality is listed in Table 4. Excluding treatment-related factors, ten factors were included in the univariate analysis. Six factors were significantly associated with CSS including timing, distribution, number, maximum tumor size, mGPS, and CEA. On multivariate analysis, factors with a *P* value of <0.05 in the univariate analysis were included. Multivariate analysis revealed a significant association between cancer-related postoperative mortality and mGPS (HR 1.595; 95 % CI 1.156–2.201; *P* = 0.004) and CEA (HR 2.044; 95 % CI 1.366–3.058; *P* = 0.001).

**Table 4:** Univariate and multivariate analysis in relation to cancer-specific death

Variable	Univariate analysis			Multivariate analysis		
	Hazard ratio	95 % CI	*P* value	Hazard ratio	95 % CI	*P* value
Age (<65/≥65 years)	1.026	0.736–1.430	0.879	–	–	–
Gender (F/M)	0.884	0.627–1.246	0.482	–	–	–
Site of primary lesion (colon/rectum)	1.042	0.746 1.456	0.809	–	–	–
Timing (metachronousmeta/synchronous)	1.585	1.130–2.224	0.008	1.296	0.875–1.921	0.196
Distribution (unilobular/bilobular)	1.647	1.182–2.296	<0.001	0.951	0.584–1.551	0.842
No. of tumors (<2/≥2)	1.788	1.260–2.537	0.001	1.528	0.933–2.504	0.092
Maximum tumor size (<3/≥3 cm)	1.496	1.069–2.093	0.019	1.038	0.693–1.557	0.855
Pathologic differentiation (well differentiated/other)	0.982	0.691–1.394	0.917	–	–	–
CEA (<30/≥30 ng/L)	2.117	1.498–2.991	<0.001	2.044	1 366–3.058	0.001
mGPS (0/1/2)	1.611	1.202–2.159	0.001	1.595	1.156–2.201	0.004

*CI* confidence interval, *CEA* carcinoembryonic antigen, *mGPS* modified Glasgow prognostic score

## Discussion

The present retrospective study analyzed individual clinical data for 343 patients who underwent hepatectomy among a pure cohort of patients with CRLM. Our results demonstrate the prognostic value of the mGPS for CRLM. Multivariate analysis revealed a significant association between CSS and mGPS and CEA. A few studies have evaluated the GPS in patients undergoing liver resection for CRLM; however, to our knowledge, this is the first study to investigate the usefulness of the mGPS. There was no difference in DFS among patients with mGPS-0, -1, and -2 (*P* = 0.2125), and patients with mGPS-0 had a smaller number of liver recurrences, resulting in a much lower rate of recurrence resection than in patients with mGPS-1 and 2.

Many studies have reported that elevated CRP levels are indicative of a poor outcome in a variety of cancers.^16^–^18^ For example, Wong et al.^20^ reported that an elevated preoperative CRP is a predictor of poor outcome in patients undergoing curative resection for CRLM Ishizuka et al.^31^ reported that in multivariate analysis, elevated CRP levels as well as the number of metastatic tumors were associated with cancer-specific death. On the other hand, hypoalbuminemia is often observed in patients with advanced cancer and is usually regarded as a good index for malnutrition and cachexia. In patients with colorectal cancer, hypoalbuminemia was reported to be associated with a poorer outcome.^30^,^36^

The GPS, which is based on both serum elevation of CRP and hypoalbuminemia, may enable a better appreciation of effects of the tumor on both ongoing systemic inflammation and malnutrition. The GPS was introduced to predict the prognosis of patients with advanced neoplasms.^21^,^25^,^29^,^30^,^37^ The GPS was recently modified on the basis of evidence that hypoalbuminemia in patients without an elevated CRP concentration has no significant association with CSS.^30^

At present, only two studies have investigated the usefulness of the GPS for postoperative death of patients undergoing curative resection for CRLM. One study revealed that in multivariate analysis, a GPS of one or two and three or more liver metastases were independent prognostic factors among 63 patients.^32^ The other study revealed that the GPS was able to classify 93 patients with resectable CRLM into three independent groups and that GPS was associated with postoperative cancer-related death in univariate analysis, but not in multivariate analysis.^31^ To date, most series on the value of GPS for patients undergoing liver resection for CRLM have been limited by small sample sizes. The sample size of the present study was relatively large. Our results revealed that a higher mGPS was associated with poorer survival in patients undergoing liver resection for CRLM, which is accordance with results of previous studies evaluating the prognostic value of the mGPS in colorectal and other cancers.

Although there were no significant differences between mGPS and timing (synchronous/metachronous), there seemed to be a trend toward higher mGPS in the synchronous group. These may influence aggressive biological behavior of synchronous liver metastases.

The clinical risk score (CRS), a score from 0 to 5 based on five preoperative variables, had been developed as useful prognostic scoring system and this system has been validated at other institutions.^38^–^40^ In our series, CRS high (≥3) was not significantly associated with CSS in univariate analysis (HR 0.809; 95 % CI 0.506–1.294; *P* = 0.377). Neoadjuvant chemotherapy was administered to 69 (20.1 %) patients and chemotherapy has changed some variables such as tumor size and CEA. Therefore, our study might not demonstrate the usefulness of the CRS.

Although our results demonstrated the prognostic value of the mGPS, Kaplan–Meier analysis and the log-rank test demonstrated no differences in DFS among patients with mGPS-0, -1, and -2 (*P* = 0.2125). Two previously reported studies did not investigate the relationship between the GPS and DFS. Several recent studies on the pattern of recurrence after hepatectomy have been reported. Hill et al.^41^ reported that patients with liver-only or lung-only recurrence had a better prognosis compared with patients with other patterns of recurrence. D’Angelica et al.^42^ reported that the recurrence pattern and resection of the recurrence were independently associated with survival from the time of recurrence in multivariate analysis. Patients with recurrence in multiple sites reportedly had a shorter survival time than patients with recurrence in a single site; furthermore, patients with resected recurrences, regardless of site, survived longer than those who did not undergo resection. In our series, recurrence patterns exhibited no differences among patients with mGPS-0, 1, or 2. Patients with mGPS-0 had much less liver recurrence and underwent much fewer recurrence resections than did patients with mGPS-1 and 2. These differences in the patterns of recurrence and proportion of resections of recurrence according to the mGPS may affect CSS.

Patients with mGPS-1 and 2 have an elevated CRP level. An elevated CRP level may be indicative of a favorable environment for the establishment and growth of distant metastases.^20^ In cell culture experiments, CRP was found to inhibit apoptosis of carcinoma cells, thereby directly regulating tumor cell growth and survival.^43^ The serum level of vascular endothelial growth factor, an angiogenic factor, is increased in the presence of an elevated CRP level.^44^ Angiogenesis plays an important role in tumor growth and is associated with a poor outcome in patients with gastrointestinal tumors.^45^,^46^ An elevated CRP level may accelerate tumor aggressiveness and reflect the tumor recurrence pattern. Therefore, patients with mGPS-1 and 2 may develop recurrences of multiple liver metastases in the remnant liver.

It is known that liver resection is the only treatment that can be used to cure CRLM.^47^,^48^ Although the 5-year survival for patients with mGPS-2 was more than 40 %, these patients survived shorter than those with mGPS-0 (*P* = 0.0040). The number of patients with mGPS-2 was relatively small and no patient had received neoadjuvant chemotherapy. We think that the mGPS may be useful to select patients with poor prognosis. Although further study will be needed, additional treatment such as neoadjuvant or adjuvant chemotherapy or immunonutritional support may be indicated for patients with mGPS-2.

Potential limitations of the present study are that was retrospective in nature and involved one university-affiliated hospital. There were significant differences between mGPS and patient characteristics such as age, maximum tumor size, and preoperative CEA level. Therefore, a large-scale prospective validation study is needed to confirm the results.

In conclusion, our results revealed that a higher mGPS was associated with multiple aggressive patterns of liver recurrence and poorer survival in patients with CRLM. The preoperative mGPS is a simple and useful prognostic factor for postoperative survival in patients undergoing liver resection for CRLM.
